# Impact of Data Processing and Antenna Frequency on Spatial Structure Modelling of GPR Data

**DOI:** 10.3390/s150716430

**Published:** 2015-07-08

**Authors:** Daniela De Benedetto, Ruggiero Quarto, Annamaria Castrignanò, Domenico A. Palumbo

**Affiliations:** 1 Dipartimento di Scienze della Terra e Geoambientali, University of Bari, Aldo Moro, Bari 70125, Italy; E-Mail: ruggiero.quarto@uniba.it; 2 Consiglio per la Ricerca in Agricoltura e l’Analisi dell’Economia Agraria, Unità di Ricerca per i Sistemi Colturali degli Ambienti Caldo-Aridi (SCA), Bari 70125, Italy; E-Mails: annamaria.castrignano@entecra.it (A.C.); domenico.palumbo@entecra.it (D.A.P.)

**Keywords:** ground-penetrating radar, amplitude maps, antenna frequency, data processing, geostatistical modelling

## Abstract

Over the last few years high-resolution geophysical techniques, in particular ground-penetrating radar (GPR), have been used in agricultural applications for assessing soil water content variation in a non-invasive way. However, the wide use of GPR is greatly limited by the data processing complexity. In this paper, a quantitative analysis of GPR data is proposed. The data were collected with 250, 600 and 1600 MHz antennas in a gravelly soil located in south-eastern Italy. The objectives were: (1) to investigate the impact of data processing on radar signals; (2) to select a quick, efficient and error-effective data processing for detecting subsurface features; (3) to examine the response of GPR as a function of operating frequency, by using statistical and geostatistical techniques. Six data processing sequences with an increasing level of complexity were applied. The results showed that the type and range of spatial structures of GPR data did not depend on data processing at a given frequency. It was also evident that the noise tended to decrease with the complexity of processing, then the most error-effective procedure was selected. The results highlight the critical importance of the antenna frequency and of the spatial scale of soil/subsoil processes being investigated.

## 1. Introduction

The assessment of soil water content (SWC) variation on both spatial and temporal scales is fundamental in many research areas and applications, such as land use planning, irrigation management, ecological and hydrological modelling. A great deal of research has gone into the development of novel SWC techniques capable of providing measurement of a physical variable that is a surrogate for SWC across a wide range of spatial scales. Reviews of soil moisture measurement techniques are given by Robinson *et al.* [1] and Vereecken *et al.* [2], with the aim to identify several emerging methods and technologies from geophysics. Geophysical methods provide a low cost and noninvasive way of gathering a large amount of information on various physical soil properties. In agriculture, the use of these methods was largely motivated by the need for reliable, quick and easy measurements of soil parameters at field and landscape spatial extents. Ground Penetrating Radar (GPR) is one of the geophysical techniques, currently used in agricultural research and application to monitor shallow soil water content [3,4,5,6]. A GPR system consists of an arrangement of antennas that can emit and receive electromagnetic pulses in the radar frequency range of 1 MHz to a few GHz. These pulses propagate through low-loss materials until they are reflected or diffracted by interfaces and objects that exhibit contrasts in the electric permittivity (ε) [7]. A review of the numerous laboratory studies, as provided in Knight [8], shows that the dominant factors controlling ε of a material are: (1) the volume fractions and dielectric constants of the individual components; (2) the geometrical arrangement or distribution of the components; and (3) the physical and/or chemical interactions between components. Conversely, given the large contrast between ε of water (81) and that of air (1) and of commonly occurring solid components (5–12), it is often assumed that variation in ε is primarily due to variation in water content. However, the development and acceptance of GPR as a soil water content sensor is still limited by the cumbersomeness of its application in field conditions, due to the complexity of data acquisition and processing.

In particular, processing procedures are necessary to compensate or minimize undesirable effects and enhance the radar images of subsoil. Filtering processes are very time consuming, requiring automation and a well-designed, conceptually rigorous filter. Several publications have described the use of 2-D and 3-D GPR images and, the quality of processed GPR image was generally evaluated visually and qualitatively according to the subjective ability of the operator to detect different interfaces or reflections. Moreover, the application of signal processing may alter drastically some characteristics of the the raw signal data. In this work the selected characteristic being investigated is the amplitude of signals reflected by any surface. The hypothesis is based on the facts that the resulting amplitude of a radar wave depends not only on intrinsic attenuation, mostly controlled by electrical conductivity, but also on reflection coefficient between layers with different dielectric properties. The amplitude will then provide information directly related to subsurface changes of the dielectric properties affecting the hydraulic properties [9,10,11]. In particular, enveloped amplitude is associated with the reflection strength of the signal, so that a large value of amplitude may indicate major changes in subsurface layers and/or lower attenuation through the layer of soil crossed by radar waves. Generally, the amplitude values were inversely proportional to water content.

Data processing may affect the spatial dependence of GPR data. In particular, some researchers have studied how the geostatistical structure of surface GPR reflection data was affected by different data gains and migration for a single radar section [11]. They observed that the sedimentary materials and their geometrical arrangement affected spatial structures of radar data but geostatistical models of radar reflection amplitudes were a function of data processing and signal frequency. Another researcher [12] used geostatistical tools in order to characterize spatial variations of the data and their resolution, and to filter out the spatial components identified as noise.

In this study, a series of data processing sequences was applied to the recorded data and after each processing sequence, and the amplitude envelope of radar signal was calculated. It is important to note that all GPR data used in this study are displayed in “time slice” (or depth slice) maps [13], a way of displaying the data in horizontal maps of recorded reflection amplitudes. The radar processing was kept to a minimum, in order to preserve the original amplitude values. GPR response, for each applied data processing sequence, was described and the approaches were compared through variography analysis and traditional statistical analysis.

The objectives were: (1) to investigate the effects of data processing on radar signal; (2) to select a quick and error-effective data processing for detecting subsurface features. The data analysis was aimed to quantify measurement error in the data submitted to different processing procedures. Finally, because the spatial structures of GPR data can exhibit marked dependence on antenna frequency, a comparison among different frequencies (250, 600 and 1600 MHz) was performed using variography techniques aimed (3) to estimate measurement error and to establish an objective criterion for selecting the most suitable antenna frequency to investigate a complex system such as the agricultural soil.

## 2. Experimental Section

### 2.1. Description of the Field Site and Mapping Protocol

**Figure 1:**
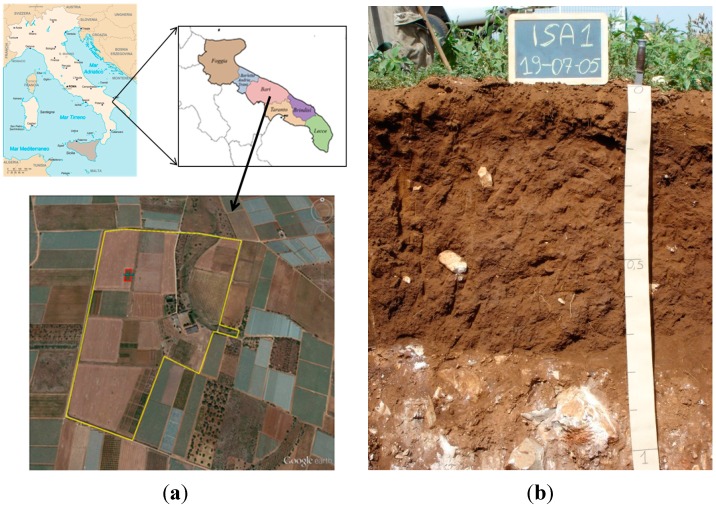
(**a**) The experimental farm (yellow polygon) located in Apulia region (south-eastern Italy) and the experimental test plot delimited by a red rectangle [18]; (**b**) Pedological pit surveyed in the study area [Reproduced with permission from De Benedetto *et al.*, Geoderma; published by Elsevier, 2012, DOI:10.1016/j.geoderma.2011.05.005].

The experiment was conducted at the “Maria Elisa Venezian Scarascia” farm, one of the experimental farms of the Italian Agricultural Research Council (CRA), located in Rutigliano-Bari (40°59′48.25″ N, 17°02′02.06″ E), in south-eastern Italy (Figure 1). The test plot selected for this study was bare soil of 40 m × 20 m size (red rectangle in Figure 1). The study area is located in the Murgia Plateau, characterized by homogeneous sequence of calcareous and dolomitic rocks. Limestones have quite low porosity but are usually fractured and affected by karst dissolution. The soil is classified as fine, mixed, superactive, thermic Typic Haploxeralfs, according to the Soil Taxonomy [14], and as Cutanic Luvisol (Hypereutric, Profondic, Clayic, Chromic), according to the WRB [15]. Soil texture is mainly clayey with the clay content ranging from 30% to 60% by weight increasing in depth and with high content of gravel (15% by weight). The coarse soil mineral fraction increases in volume in the neighbourhood of the outcropping bedrock. The soil depth, as it was also revealed by the pedological survey [16], is rarely deeper than 0.60–1 m, owing to the occurrence of shallow bedrock (Figure 1b). Since high contents of clay may strongly attenuate the signal, a preliminary ERT survey was carried out which allowed us to estimate the resistivity of soil (values between 25 and 60 Ωm) and radar energy attenuation (values between 6 and 14 dB/m). These values show that Ground Penetrating Radar can be effectively and reliably used in this study area. Additional CMP measurements, carried out through a bistatic GPR system with central frequency of 450 MHz, were used to determine velocity profiles [17].

The bare plot was surveyed with two different monostatic GPR equipments along transects N–S and E–W oriented and about 1 m apart using the common-offset method: one, a Noggin 250 MHz (Sensors & Software Inc., Mississauga, ON, Canada), operates with a central frequency of 250 MHz and spans a 3 dB bandwidth from 125 to 375 MHz and with shielding front to back >20 dB and the other, a RIS 2 k-MF Multifrequency Array Radar-System (manufactured by IDS Ing, Pisa, Italy), with two central frequencies of 600 MHz (bandwidth from 300 to 900 MHz) and 1600 MHz (bandwidth from 800 to 2400 MHz). The 250 MHz GPR system acquired the data using a time window of 100 ns with a temporal sampling interval of 0.2 ns and spacing between the traces of 0.05 m collected after 16 stacked radargrams with a performance factor of 172 dB. The 600 and 1600 MHz GPR system worked with a time window of 60 ns and a temporal sampling interval of 0.05 ns; successive traces were collected every 0.024 m with a performance factor of 172 dB. The coordinates of the initial and final positions of GPR transects were recorded using a differential global positioning system with planimetric centimeter accuracy. Detailed discussions of the fundamental principles of GPR can be found in the publications by Daniels *et al.* [19] and Davis and Annan [7].

### 2.2. GPR Data Processing

**Figure 2:**
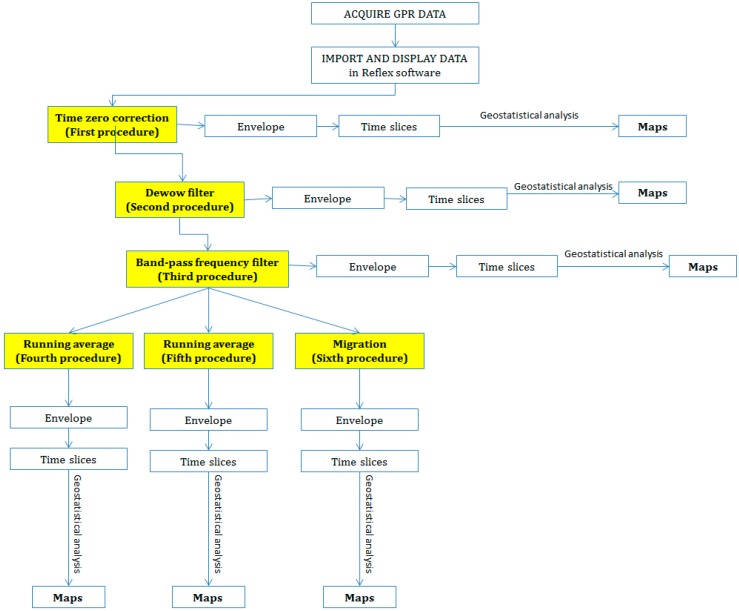
A schematic representation of GPR data analysis.

All GPR data were processed with ReflexW Software [20] and six different data processing sequences were applied to all radar sections (Figure 2): 1° procedure: Time zero correction; 2° procedure: Time zero correction and dewow filtering; 3° procedure: Time zero correction, dewow filtering and band-pass frequency filter; 4° procedure: Time zero correction, dewow filtering, band-pass frequency filter and running average on a defined number of traces covering 0.5 m; 5° procedure: Time zero correction, dewow filtering, band-pass frequency filter and running average covering 1 m; 6° procedure: Time zero correction, dewow filtering, band-pass frequency filter and migration.

Detailed descriptions of the above procedures can be found in the historical publications by Reynolds [21] and Cassidy [22].

The signal processing parameters applied to the data are summarized in Table 1. The computer time varied for each procedure, increasing with the complexity of processing. No amplitude gain functions were applied to the data because they are more likely to destruct relative amplitude information [23]. After each procedure, the instantaneous amplitude or envelope of data was calculated using a quadrature filter (Hilbert transformation). This filter is traditionally used to transform a real-value signal to an analytic signal, *i.e.*, a signal that has no negative frequency components. In practice, quadrature filters give an estimation of the energy distribution of the traces [24]. It is a measure of the reflectivity strength and is proportional to the square root of the total energy of the received signal at a given time instant. Envelope can then give an overview of the distribution of the different types of reflectors present in the subsoil and then permits the construction of a structural model of subsoil.

**Table 1:** GPR processing procedures and parameters used for each frequency with ReflexW Software.

Process	250 MHz	600 MHz	1600 MHz
**Time zero**	22.76 ns	2.56 ns	3 ns
**Dewow**	4 ns	1.6 ns	0.6 ns
**Bandpass frequency**	100–200–300–400 MHz	300–550–650–900 MHz	1300–1550–1650–1900 MHz
**Simple running average**	10 traces 20 traces	20 traces 40 traces	20 traces 40 traces
**Migration**	Mean velocity = 0.06 mns^−1^ up to 10 ns Mean velocity = 0.1 mns^−1^ after 10 ns	Mean velocity = 0.06 mns^−1^ up to 10 ns Mean velocity = 0.1 mns^−1^ after 10 ns	Mean velocity = 0.06 mns^−1^

One of the most impressive ways of displaying GPR data is in horizontal maps that allow easy visualization of location, depth, size and shape of the radar anomalies buried in the ground. The maps can be created at various time levels within a data set to show radar information at a specified time (depth) across a surveyed site. Therefore, enveloped amplitude maps (time slices) were built averaging the amplitude (or the square amplitude) of the radar signal, expressed in digital number (DN), within overlapping time windows of width Δt. Typically Δt must be of the order of the dominant period of the antennas (4 ns, 2 ns and 1 ns for 250, 600 and 1600 MHz antennas, respectively), because GPR reflections are normally taken over a time window of a microwave pulse length. The total time interval was of 20 ns for 250 and 600 MHz because this time was comparable with the depth of soil (at 0–0.30 m depth), and of 5.5 ns for 1600 MHz because of the attenuation of radar signal.

For visual representation and interpretation of radar sections, reported in order to support the interpretation of the relationship observed by statistical and geostatistical analysis, standard GPR data processing was performed including: time zero correction, dewow, automatic gain control (AGC), background removal and bandpass filter.

### 2.3. Geostatistical Methodology

The multivariate dataset, consisting of GPR time/depth slices after processing (Figure 2), was interpolated using multiGaussian cokriging [25]. This approach is based on a multiGaussian model which requires a prior Gaussian transformation of each attribute into a Gaussian shaped variable with zero mean and unit variance. Such a transformation procedure, known as Gaussian anamorphosis, consists in determining a mathematical function which transforms a variable with a Gaussian distribution into a new variable with any distribution [26,27]. Gaussian anamorphosis was performed by using an expansion in Hermite polynomials restricted to a finite number of terms [27].

As for variogram fitting, a Linear Model of Coregionalization (LMC) was applied. LMC, developed by Journel and Huijbregts [28], considers all the studied variables as the result of the same independent physical processes, acting at *N_s_* spatial scales, *u*. The *n*(*n* + 1)/2 simple and cross-semivariograms (γ) of the n variables are modelled by a linear combination of *N_s_* semivariograms standardized to unit sill *g_u_*(h), which are assumed to be the same for all variables at a given spatial scale *u*. Using the matrix notation, the LMC can be written as:

$$\Gamma \left(h\right)=\overset{N_{S}}{\underset{u=1}{\sum }}B^{u}g^{u}\left(h\right)$$

where **Γ**(**h**) = [γ*_ij_*(**h**)] is a symmetric matrix of order *n* × *n*, whose diagonal and out of-diagonal elements represent simple and cross-semivariograms, respectively for lag **h**; **B***^u^* = [*b^u^_ij_*] is called coregionalization matrix and it is a symmetric positive semi-definite matrix of order *n* × *n* with real elements *b^u^_ij_*, which represent the sills of the direct (if *i* = *j*) and (cross-) variograms *ij* (for *i* ≠ *j*) at a specific spatial scale u among the selected *N_s_* scales. The model is authorized if the mathematical functions *g^u^*(**h**) are mathematically authorized semivariogram models under the constrain of positive semi-definiteness of each **B***^u^*, being assumed to represent a variance-covariance matrix at the spatial scale u. Fitting of LMC is performed by weighed least-squares approximation under the constraint of positive semi-definiteness of the **B***^u^*, using an iterative approach developed by Rivoirard [29]. Goodness of fitting was evaluated using cross-validation and in particular by calculating mean error and mean squared standardized error, which have to be close to 0 and 1, respectively.

In LMC, total variance can be decomposed into spatially structured variance at different spatial scales and spatially unstructured variance (nugget). The nugget effect represents unexplained spatially dependent variation (microvariability at distances closer than the smallest sampling lag) or purely random variance (like measurement or sampling error). The proportion of total variation can warn us of large measurement errors or of the need to sample more densely, or both [30] and for this reason, it can be used as indicator of map quality.

The GPR data were interpolated with block cokriging in order to reduce the variability, using a regular 5 × 5 discretization of each 0.5 m × 0.5 m block; finally the estimates were back-transformed to amplitude of the radar signal. Geostatistical procedures were separately applied to the data of each antenna frequency, by using the software package ISATIS^®^, release 2014 [31]. After interpolation, to eliminate the noise due to variable energy of the transmitted signal, the estimated amplitudes were normalised by using, as reference, the first time slice mainly related to the radar waves in air.

### 2.4. Comparison between the Maps

In order to make the comparison among the maps more objective and to choose the most efficient GPR processing in a less subjective way, the spatial association between paired GPR maps was calculated using confusion matrix and k statistic. The efficiency of data processing was evaluated in terms of maps quality and computer time consuming. The maps were preventively classified in ten isofrequency classes of radar signal amplitude and the classes were then compared in pairs by using a two-enter table (confusion matrix). Each cell of the matrix gave the absolute frequency of the occurrence of the two corresponding classes [32]. The overall consistency, which is a measure of the spatial association between two maps, was computed as the proportion of the total number of observations along the main diagonal of the contingency matrix. Weighted kappa coefficient, introduced by Cohen [33], measures the inter-classification agreement and equals 0 when the agreement is due to chance and +1 when there is perfect agreement. When kappa is negative, it is assumed no agreement. Besides kappa coefficient, its confidence limits [34] were computed. The approach was implemented with the FREQ procedure of the SAS/STAT software package [35].

## 3. Results and Discussion

### 3.1. First Visual Interpretation

**Figure 3:**
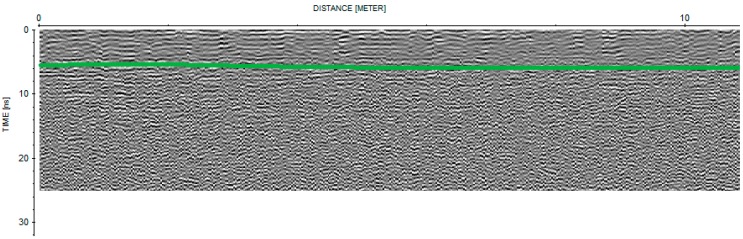
A part of GPR common-offset radar section acquired along a N–S oriented profile (Profile 14) with the 1600 MHz antenna. The depth is represented by time and the green line represented a discontinuity.

**Figure 4:**
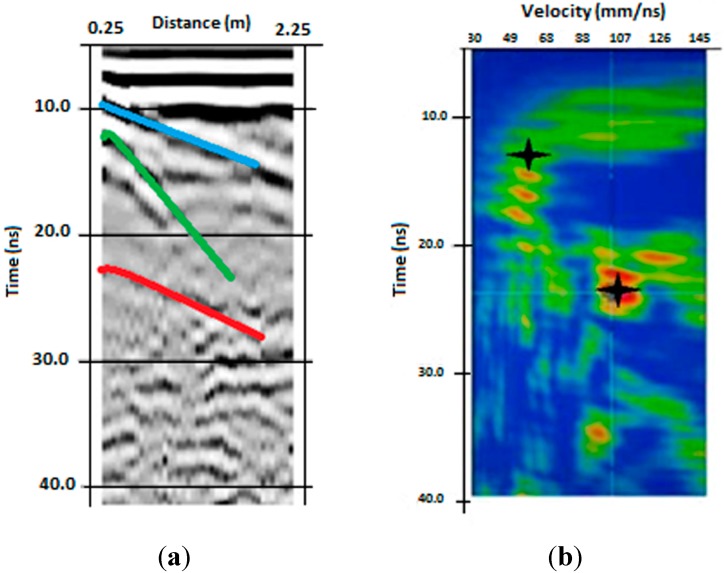
Example of CMP measurements: time section after a basic processing (**a**) and velocity spectrum (**b**). In (**a**) the colored lines represent the air wave (light blue), the second reflection (green) and the bedrock reflection (red). In (**b**) the colors, from blue to red, are proportional to the amplitude of stacking velocity and the crosses represent the attributed average velocities.

From the visual inspection of GPR radar sections and CMP data, three main layers were synthetically disclosed: a first layer at time ranging between about 3 ns to about 5 ns (0.09–0.15 m depth, considering a velocity of 0.06 mns^−1^), visible only in the radar sections acquired with 1600 MHz frequency (an example is reported Figure 3), a second layer between 10 ns and 14 ns (0.3–0.4 m depth with a velocity of 0.06 mns^−1^) and a third layer between 20 ns and 22 ns (0.6–0.66 m depth with an average velocity of 0.1 mns^−1^), detectable from all the radar sections acquired with 250 and 600 MHz frequencies and from CMP data (example of CMP data is reported in Figure 4). For convenience these reflections are referred to as the “first”, “second” and “third” reflection, respectively. The “first” and “second” reflected layers may be related to interfaces in the soil, probably due to shallow ploughing or soil compaction caused by tractor passage and/or tillage. Conversely, the “third” reflected layer was ascribed to the soil-bedrock interface because of its wide amplitude, denoting a strong electromagnetic contrast, and on the basis of pre-existing pedological profile (Figure 1b). The bedrock reflection was generally characterized by marked roughness (more or less evident in different parts of the site) and many anomalies of various types (hyperbolic signals) were observed in the overlying soil layer. The radar sections of the different antennas showed varying features in terms of resolution, and it was preferred not to select only one antenna because all antennas jointly captured the scale-dependent variation of soil/subsoil. Moreover, the propagation velocity was equal to 0.06 mns^−1^ up to 10 ns and for longer times the average velocity was 0.1 mns^−1^, assuming a subsoil model with horizontal stratification and constant lateral velocity (Figure 4b).

### 3.2. Statistical Analysis of Data

All the time slices showed a sensible attenuation of signal at about 20 ns (about 0.90 m depth) for 250 and 600 MHz frequencies and at about 5.5 ns (about 0.15 m) for 1600 MHz frequency; therefore, all GPR data coming from the longer travel times (deeper depths) will not be treated from now onwards.

**Figure 5:**
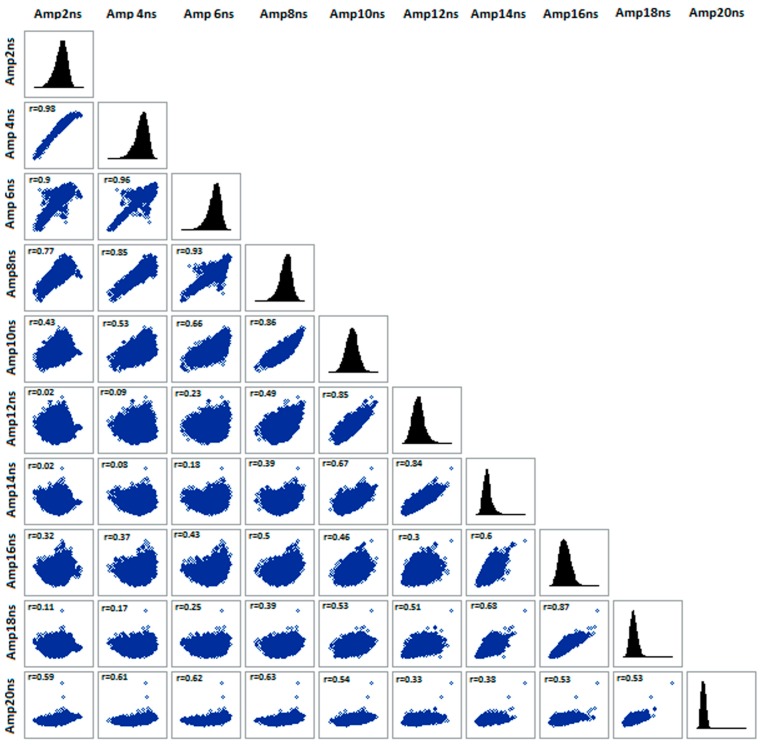
Graph of correlation matrix for GPR data acquired with 250 MHz antenna and processed with third processing procedure (time zero correction, dewow filter, bandpass filter and envelope). Person’s correlations (r) are reported and all coefficients are significant at 0.05 probability level.

Statistical analysis highlighted that GPR data at any frequency and for all procedures showed clear departure from normal distribution, however the addition of further steps in the processing (after the third procedure) caused a more symmetric distribution. In addition, Person’s correlations allowed you to disclose the main reflections observed visually in the radar sections and to evaluate the strength of spatial association between the data at the different depths as function of the processing procedure. The GPR amplitudes at the different times were strongly correlated within an interval of 10–14 ns, for 250 MHz antenna (mainly evident in the data processed with the third procedure, as reported in Figure 5). This time interval may be related to the “second” reflection, which was not detectable visually in the radar sections but only in CMP data. The correlation coefficient showed a discontinuity in the range 10–12 ns for the 600 MHz antenna at the corresponding depth range of 0.3–0.36 m, not evident in the radar sections, which may be due to agricultural tillage. On the contrary, the correlations between the time slices corresponding to the 1600 MHz antenna indicated the presence of a discontinuity at 3.5–4 ns (corresponding at 0.1–0.12 m depth), not detectable in the other antennas, which may be due to the presence of organic residuals in the first ten centimeters.

Furthermore, from the statistical analysis, the third procedure seems to have achieved a good trade-off between the quality of the signal at any travel time (the data distribution was quite normal and with few outliers, as a consequence of the band-pass filter, and the correlation coefficients increased so improving the signal in depth) and the computer processing time demand (the computer time for the fourth procedure was longer) for any frequency.

### 3.3. Geostatistical Results

#### 3.3.1. Visual Interpretation of the Estimated Maps

All GPR data at any time interval for each antenna were correlated so to justify the application of a multivariate approach. The data, being generally skewed, were previously transformed into standard Gaussian variables and a linear isotropic model of coregionalization (LMC) was fitted to the all experimental direct and cross-variograms separately for each processing procedure and each antenna.

Independently of the kind of processing, the basic structures were identical for any antenna, showing that the procedures did not alter the intrinsic spatial structures. Geostatistical modelling of radar amplitude was affected by data processing only for the estimated parameters of nugget effect and partial sill. The LMCs consisted of the following basic structures: (1) for 250 MHz: Nugget effect, Spherical model (Range = 3 m) and Spherical model (Range = 12 m); (2) for 600 MHz: Nugget effect, Spherical model (Range = 1.2 m) and K-Bessel model (Range = 10 m); (3) for 1600 MHz: Nugget effect, Spherical model (Range = 0.5 m) and Exponential model (Range = 12 m). The goodness of LMC fitting was satisfactory because mean error was quite close to 0 and the mean square standardised error was almost 1 for all data sets.

**Figure 6:**
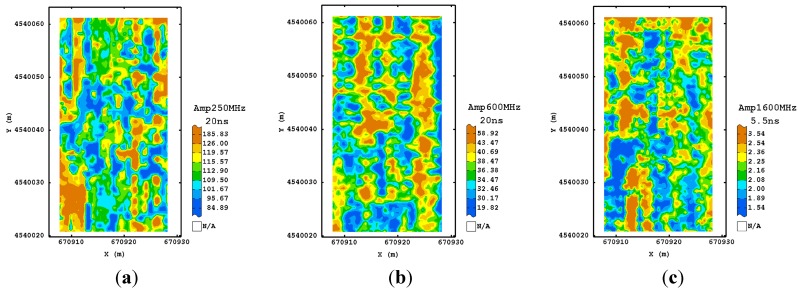
Maps of estimated amplitude for 250 MHz (**a**); 600 MHz (**b**) and 1600 MHz (**c**) frequencies using the first processing procedure corresponding to their maximum depth investigated. (Colour scale uses iso-frequency classes).

As regards the first procedure at 250 MHz frequency, the visual inspection of the estimated normalized amplitude maps at the different times showed that the processing did not modify sensibly the signal at any depth, preserving the raw information. However, the signal in depth was not usable because of the high attenuation (Figure 6a). For both 600 and 1600 MHz antenna, the maps were very noisy and did not showed well defined spatial structures (Figure 6b,c).

**Figure 7:**
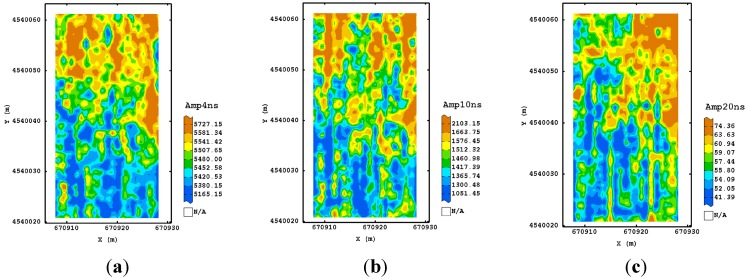
Maps of estimated amplitude for 250 MHz antenna frequency using the third processing procedure corresponding to 4 ns (**a**); 10 ns (**b**) and 20 ns (**c**). (Colour scale uses iso-frequency classes).

For the second processing, the maps at 250 MHz frequency and corresponding to the longer time slices (16–18 ns) appeared well structured and more associated with the ones observed in the shallower depths. Therefore, the addition of a further step in the GPR processing improved the signal, recovering more information from the deeper depths. This characteristic was not valid for the other frequencies, suggesting that no improvement was obtained with the addition of dewow, as expected for the higher sampling frequencies used. As for the third procedure, all the maps of the estimated amplitude at different times (Figure 7) displayed some consistency up to the deeper depth. A tendency for higher values of amplitude was detectable along the north-eastern and south-western sides of the plot.

**Figure 8:**
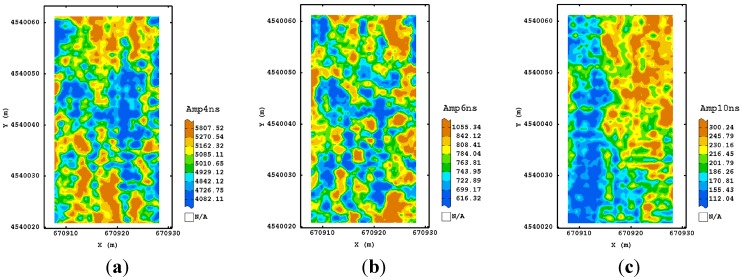
Maps of estimated amplitude for 600 MHz antenna frequency using the third processing procedure corresponding to 4 ns (**a**); 6 ns (**b**) and 10 ns (**c**) as an example. (Colour scale uses iso-frequency classses).

**Figure 9:**
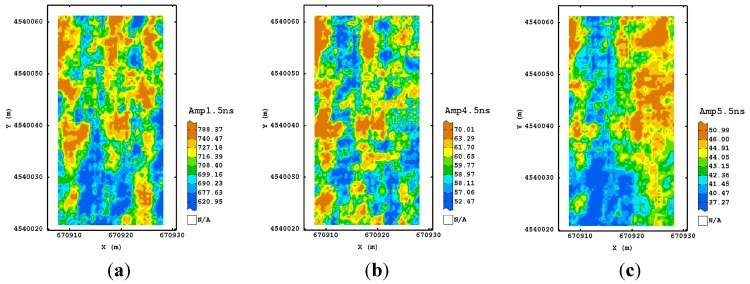
Maps of estimated amplitude for 1600 MHz antenna frequency using the third processing procedure corresponding to 1.5 ns (**a**); 4.5 ns (**b**) and 5.5 ns (**c**) as an example. (Colour scale uses iso-frequency classes).

An improvement was also visible for the maps of the other frequencies (Figure 8 and Figure 9) which looked more variable because of their finer spatial resolution. They showed a similar spatial pattern after 10 ns for 600 MHz frequency and after 5.5 ns for the 1600 MHz frequency, which consisted of two blocks along the longitudinal axis, with the eastern side characterized by higher values of amplitude. This type of processing then seems to reach a good trade-off between the quality of signal at any travel time/depth and the complexity of signal processing.

**Figure 10:**
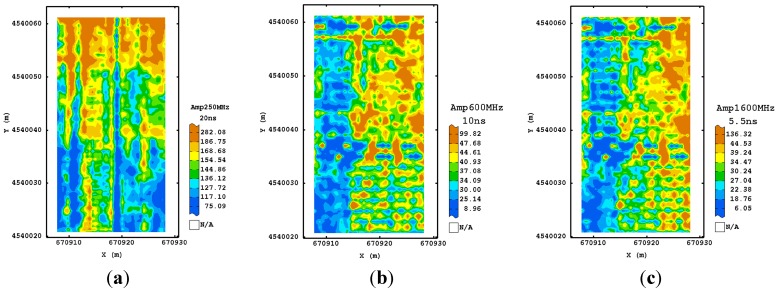
Maps of estimated amplitude for 250 MHz (**a**); 600 MHz (**b**) and 1600 MHz (**c**) frequencies using the sixth processing procedure. (Colour scale uses iso-frequency classes).

No significant improvement was observed in the maps obtained with the application of running average (fourth and fifth processing) even if the maps looked slightly more smoothed. Finally, the maps obtained with the sixth procedure were quite similar to the previous maps, though the quality has worsened for some of them, in particular for the deeper maps (Figure 10), because of the presence of quite evident artifacts. This was probably due to the assumption of a subsoil model with horizontal stratification and the use of an average velocity for all radar sections of a given frequency. In a complex environment, as the one studied, this assumption is probably violated, therefore the migration can cause errors and does not improve the quality of maps.

#### 3.3.2. Quantification of Measurement Error

**Figure 11:**
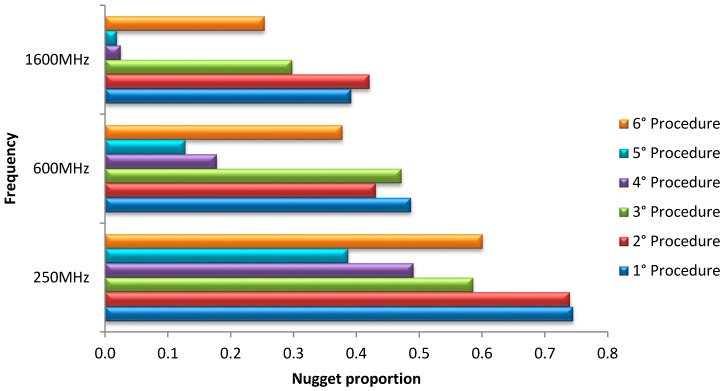
Variance proportion explained by nugget component for each used processing procedure and for each antenna.

Since the best processing should be selected on its capability of filtering spatially uncorrelated error (white noise of GPR signal, nugget effect in geostatistics), the proportion of the total variance associated with nugget component was calculated (Figure 11).

It is evident that the error has a tendency to decrease with the complexity of processing, with the exception of the last procedure, confirming in objective way most of the previous considerations.

As for 250 MHz frequency, the main differences were between the first two processing procedures and the third one (about 16%), while the successive steps did not reduce sensibly the measurement error, with the exception of the last one that caused an increase. As explained previously, a reason, is that migration relies on the knowledge of velocity; therefore, an uncertain estimation may cause error in prediction of signal. An improvement in the results may be achieved by using an accurate subsurface velocity model obtainable with CMP semblance technique, which requires a longer time of signal acquisition and processing. For the other antenna frequencies (Figure 11), the nugget proportion was lower than the one at 250 MHz frequency, due to the finer footprint. The third procedure did not reduce greatly the measurement error (13%), as much as for the fourth and fifth procedures (about 40%–45% for 600 MHz antenna and about 35%–37% for 1600 MHz antenna). These last procedures (for all frequencies) showed the lowest values of nugget proportion, but no significant improvement was observed in the maps. The sixth procedure, also in this case, caused an increase of nugget proportion compared with the ones of the 3–5 procedures, and the quality of maps was then worse. Therefore, also in the light of the previous considerations, the third processing could be considered as optimal in terms of map quality and complexity of signal processing.

### 3.4. Comparison among the Different Processing Procedures: Results

For each frequency, all maps corresponding to the different types of processing were compared with the one of the third processing procedure, assumed as reference. In the interest of conciseness, only the results at 250 MHz frequency will be presented. As regards the shallower depth, the maps corresponding to the first two procedures generally showed lower overall accuracy and weighted kappa coefficient, which is indicative of poor spatial association with the reference map. The use of these procedures then changed the signal structure in the shallower layers, though at a visual inspection the maps appeared very similar. Moderate association (about 50%) was obtained with the fourth procedure, indicating no actual improvement. Moreover, the poor agreement (about 20%) with the procedure after migration confirmed the result obtained through a visual comparison of the maps. On the contrary, the overall accuracy for the maps corresponding to 10 ns (Table 2) was higher for the first two procedures and the kappa coefficients were significantly greater than 0 and indicative of a moderate spatial agreement. The use of additional steps of processing (from the fourth to sixth procedure) reduced the overall accuracy. Finally, the results related to the deeper time slices indicated very weak agreement, demonstrating the modification of signal in depth with the use of these additional steps.

In conclusion, the traditional statistical analysis allowed you to objectively state that the spatial association with the map of the third procedure decreased with the complexity of processing. However, geostatistics with nugget effect estimation provided a tool of testing the quality of maps and then a criterion for selecting the optimal processing.

**Table 2:** Overall accuracy and kappa coefficient with its 95% Lower and Upper confidence limits between the time slices related to 10 ns for 250 MHz frequency obtained with different procedures and compared with the reference procedure (third procedure).

Amp10 ns	Overall Accuracy	Weighted Kappa Coefficient		
**3rd procedure–1st procedure**	0.58	0.83	95% Lower 95% Upper	0.8205 0.8407
**3rd procedure–2nd procedure**	0.60	0.85	95% Lower 95% Upper	0.8451 0.8612
**3rd procedure–4th procedure**	0.319	0.73	95% Lower 95% Upper	0.7206 0.7431
**3rd procedure–5th procedure**	0.352	0.69	95% Lower 95% Upper	0.6808 0.7059
**3rd procedure–6th procedure**	0.308	0.61	95% Lower 95% Upper	0.6012 0.6314

### 3.5. Comparison among the Different Antenna Frequencies: Results

In order to investigate the response of GPR as a function of operating frequency, we restricted the geostatistical analysis only to the data processed according to the third procedure. The estimated structures (type, range, sill) of GPR data are expected to depend on the antenna frequency, since the radar signal wavelength affects spatial resolution, which is determined by the area illuminated by a GPR antenna, often referred to as the Fresnel zone or antenna footprint [7] or support in geostatistics.

The LMCs (reported in Section 3.3) revealed the presence of three basic structures at different spatial scales in the horizontal plane. The cumulative values of the eigenvalues associated with each spatial structure showed the main components of variation to be the nugget effect (0.58, 0.47 and 0.3 for 250, 600 and 1600 MHz frequencies respectively) and the structure at short range (0.25, 0.34 and 0.62 for 250, 600 and 1600 MHz frequencies respectively). For 250 MHz frequency the highest proportion of variation was associated with nugget effect, because of a wider support.

At the lowest frequency, the maps looked smoother and more continuous than at higher frequency, because they were less sensitive to small scale features and produced a LMC with longer ranges. This result was confirmed by the visual interpretation. It can be observed that the shorter range (3 m, 1 m and 0.5 m for 250, 600 and 1600 MHz frequencies, respectively) was quite close to the wavelength calculated for propagation velocity of 0.06 m/ns (2.4 m, 1 m and about 0.4 m for 250, 600 and 1600 MHz frequencies, respectively), which is directly proportional to the measurement resolution. The short-range structure was probably more sensitive to the type of equipment used, whereas the longer range was more influenced by the material properties. Some researchers obtained similar results using different antenna frequency but they asserted that they depend not only on the material and its intrinsic structure but also on data processing and signal frequency [10]. The longer-scale structure may then be related to soil texture and hydraulic properties. This issue should be further investigated by assessing and modelling the relationships of GPR data with textural and hydraulic properties [36].

## 4. Conclusions

The approach presented in this paper aims to investigate the effects of data processing on radar signal and to select a data processing procedure which is quick and efficient in terms of both computer time and quality of maps. Geostatistical analysis applied to three different frequencies, stressed the critical importance of the scale of survey and the need to utilise a proper equipment to capture the scale-dependent variability of soil/subsoil.

The processing procedure, based on the instantaneous amplitude or envelope, was evaluated with statistical and geostatistical techniques. Statistical analysis allowed you to detect the major reflections of the radar signal, and other useful information not evident at visual interpretation of radar sections. Geostatistical analysis provided also a criterion to select the most efficient processing on the basis of its capability of reducing spatially uncorrelated error. The selected error-effective procedure included time zero correction, dewow filter, bandpass filter and envelope. The main limitation of the proposed procedure consisted in the extremely heterogeneous subsurface conditions of study site because the received signal was the result of multiple interactions. Further studies should be conducted to improve the interpretation of such type of data. In particular the obtained results indicated that geostatistical tools, like geostatistical component filtering [25], could be applied to reduce noise and systematic variation and produce more reliable maps of amplitude.

Signal frequency exhibited a drastic influence on the spatial structures of geostatistical modelling, because of its relation to spatial resolution. The pattern of spatial structures estimated by geostatistics can be related to both radar equipment (short range) and soil properties (long range). Disclosing which soil physical properties are mainly responsible for the observed spatial structures of GPR signal remains an interesting and challenging research topic for future investigations.
